# Field efficacy study of a novel ready-to-use vaccine against mycoplasma hyopneumoniae and porcine circovirus type 2 in a Greek farm

**DOI:** 10.1186/s40813-015-0006-x

**Published:** 2015-11-01

**Authors:** Eleni D. Tzika, Panagiotis D. Tassis, Dimitrios Koulialis, Vassileios G. Papatsiros, Tom Nell, Georgia Brellou, Ioannis Tsakmakidis

**Affiliations:** 1 Farm Animals Clinic, School of Veterinary Medicine, Faculty of Health Sciences, Aristotle University of Thessaloniki, University Campus, 54124 Thessaloniki, Greece; 2 Lagou 59, 41334 Larissa, Greece; 3 Clinic of Medicine, Faculty of Veterinary Medicine, University of Thessaly, Trikalon 224, P.O. Box 199, Karditsa, Greece; 4 MSD Animal Health, Clinical Study Team Biologicals, P.O. Box 31, 5830 AA Boxmeer, The Netherlands; 5 Laboratory of Pathology, School of Veterinary Medicine, Faculty of Health Sciences, Aristotle University of Thessaloniki, University Campus, 54124 Thessaloniki, Greece

**Keywords:** Porcilis PCV M Hyo, *Mycoplasma hyopneumoniae*, Porcine circovirus, Vaccination, Pigs

## Abstract

**Background:**

The primary objective of this study was to assess the efficacy, under field conditions, of a novel ready-to use *Mycoplasma hyopneumoniae* (M hyo) and Porcine circovirus type 2 (PCV2) combination vaccine given to piglets as one vaccination (1-shot) at 3 weeks of age. The study was carried out according to a controlled, randomised, and blinded design in a Greek pig herd with clinical M. hyo and subclinical PCV2 infection. Moreover, based on serology at the time of vaccination, the average PCV2 titre was 9.15 log_2_ and represented the level of maternally derived antibodies (MDA). In total 602 healthy suckling piglets, originating from 4 weekly farrowing batches were allocated randomly, within litters, to one of two groups. The pigs in one group were vaccinated with the test product and the other pigs were injected with saline.

**Results:**

Vaccination significantly reduced lesions of craneo-ventral pulmonary consolidation in vaccinated group [expressed as lung lesion score (LLS)] (Mixed model ANOVA: *p* < 0.0001). The mean LLS was 17.1 in the controls and 10.6 in the treatment group, respectively. The average daily weight gain (ADWG) during the finishing (54 g better in the treatment group) and whole study period (34 g better in vaccinated animals) was significantly greater in vaccinated than control pigs. The vaccinated pigs had a significant reduction of PCV2 viraemia when compared with the controls.

**Conclusions:**

The test product was considered effective in the face of average MDA, based on significantly reduced severity of LLS and PCV2 viral load, as well as improved ADWG in vaccinated versus control pigs.

## Background

*Mycoplasma hyopneumoniae* (M. hyo) and Porcine circovirus type 2 (PCV2) are two of the most important pathogens in swine. Economical impact of M. hyo and PCV2 infections in swine farms worldwide can be considered as significant [1, 2]. Enzootic pneumonia (EP) is a major clinical respiratory disease in swine [3]. It is characterized by non-productive cough, growth retardation with higher feed conversion and decreased body weight gain and craneo-ventral pulmonary consolidation. M. hyo infection along with other pathogens such as *Pasteurella multocida* and other is the aetiology of EP. Further implication of other pathogens (e.g. viral agents) in EP, result in the creation of the Porcine Respiratory Disease Complex (PRDC) [2, 4–9].

The usual method for protection against EP in swine farms is routine vaccination with various commercial vaccines either as one-shot or as two-shot administration schemes [9]. A number of previously reported studies have emphasized the benefits from such routine vaccination programmes either after intramuscular [10, 11] or intradermal administration [3, 11], against the negative impact of EP on health and productive parameters of infected pigs. However, despite the extensive vaccination efforts in the vast majority of swine farms worldwide, M. hyo remains a major problem [12].

The PCV2 has been associated with post weaning multisystemic wasting syndrome (PMWS) porcine dermatitis and nephropathy syndrome (PDNS), porcine respiratory disease complex (PRDC) and the occurrence of proliferative and necrotizing pneumonia, as well as reproductive failure and enteritis [13, 14]. All the above-mentioned conditions along with subclinical PCVD cases are described as porcine circovirus diseases (PCVD) and cause great economical losses in pig production worldwide [14]. During the past few years a number of commercially used vaccines have been the main control mechanism against PCVD in swine farms [15]. Previous reports suggest a varying but nevertheless positive outcome of vaccination against PCV2 [13, 16–18]. However, due to its transmission characteristics, the virus "stays" within pig farms for years without any further need for re-introduction due to frequent pig movements within farm compartments and the continuous renewal of susceptible animals [19].

On the other hand, the interference of PCV2 maternally derived antibodies (MDA) in the priming of piglets' immune response after vaccination is a not fully quantified fact. It has been reported that this effect depends on the level of MDA. Previous research suggests that optimal vaccination strategies against PCV2 must take into consideration the correct time of vaccination, in order to have a 3-side balance including the time needed for immune response prior to exposure to pathogens, the time needed for MDA to decline and the maturation of the piglets immune system to respond adequately [14, 17].

The aim of this study was to investigate the efficacy of a novel combination vaccine against EP and PCVD simultaneously as a one-shot vaccination at 3 weeks of age under field conditions. The assessment of the test product's efficacy under the interference of average PCV2 MDA level was also a significant research target in this study.

## Results

### Body weight and ADWG

The ADWG during the finishing and over the whole study period was significantly higher in the vaccinated group when compared with controls. Specifically, vaccinated animals showed improved mean ADWG by 54 ± 9 g/day during the finishing period and 34 ± 6 g/day during the whole trial period, in comparison to the control group. The ADWG differences among groups were not statistically significant in the nursery period. Although mean body weight among the two groups was not significantly different at three time points of the study, body weight at slaughter age was 4.9 kg greater in vaccinated animals. The mean body weight at three different time points and the adjusted mean ADWG per vaccination group and per period are presented in Table 1.

**Table 1:** Mean body weight and average daily weight gain (ADWG), by vaccination group and time point-period

Time point or period	Group	
	Porcilis PCV M Hyo (*n* ^c^ = 303)	Control (*n* = 299)
	Body weight (kg)	
One week old	2.2 ± 0.6 (*n* = 303)	2.2 ± 0.6 (*n* = 299)
Transfer to finishing	18.5 ± 3.6 (*n* = 297)	18.3 ± 3.6 (*n* = 293)
Pre-slaughter	96.6 ± 12.1 (*n* = 256)	91.7 ± 11.3 (*n* = 255)
	ADWG (g/day)^║^	
	Porcilis PCV M Hyo	Control
Nursery period	286 ± 11^a^	282 ± 11^a^
Finishing period	816 ± 16^a^	762 ± 16^b^
Overall	619 ± 9^a^	585 ± 9^b^

Mean body weight expressed as kg ± standard deviation and ADWG presented as g/day. Mean ADWG is adjusted for sow, sex, batch and initial body weight ± standard error of the mean

Level of significance (ANOVA) among superscripts : Nursery period=0.3944, Finishing period ^ab^< 0.0001, Overall ^ab^< 0.0001

^c^ *n* = number of animals

### Lung and pleuritis lesions

Vaccination with the test product significantly reduced lesions of craneo-ventral pulmonary consolidation, expressed as mean lung lesion score (LLS), from 17.1 (standard deviation: 11.1) in the control group to 10.6 (standard deviation: 9.6) in vaccinated pigs (mixed model ANOVA: *p* < 0.0001). The incidence of pleuritis lesions (score ≥1) did not differ significantly among groups [Cochran Mantel Haenszel (CMH) test: *p* = 0.0609], and it was 29 % in the controls and 21 % in the vaccinated group, respectively.

### Mortality and morbidity

A total of 34 animals (17 vaccinated and 17 controls) died or were culled during the study period. Mortality was associated with enteritis (diarrhoea) in 10 animals (5 in each group) during the nursery period and respiratory disease in 11 animals (5 in vaccinated group and 6 controls) during the growing/finishing period (with findings of fibrino-necrotizing pleuropneumonia and fibrinous polyserositis). Thirteen animals died due to other reasons (eg spine injury, or trauma).

As for morbidity (defined as the proportion of animals that received individual medication), forty-nine (49) animals in the vaccinated group (16.2 %) and 58 controls (19.4 %) received individual medication. Specifically, 17 animals from the vaccinated group and 25 control pigs showed clinical signs of gastrointestinal disease (diarrhea), while 31 treated animals and 32 control pigs showed severe respiratory signs and were treated individually. Morbidity comparison among groups did not reveal statistical differences (CMH test: *p* = 0.3031).

### Blood serum tests

**Fig. 1:**
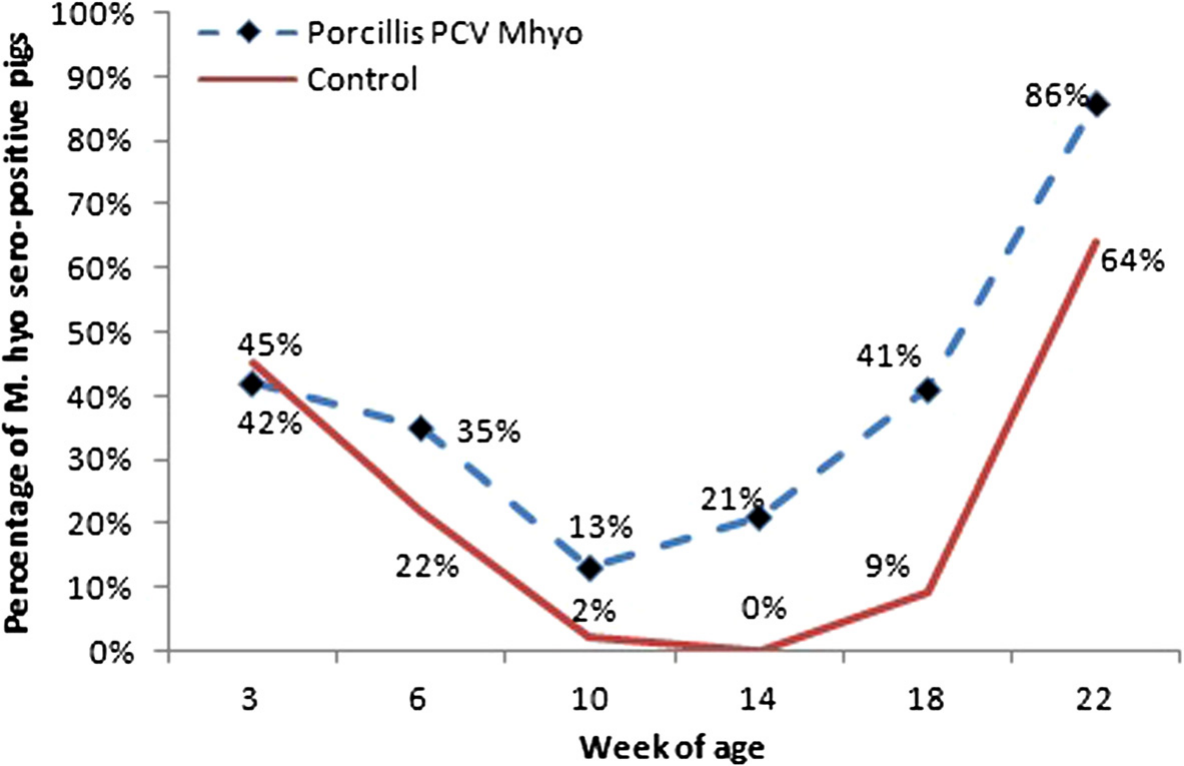
M. hyo serological results, by vaccination group and week of age legend: Statistical differences levels: Week 3: *p* = 0.5789, Week 6 *p* = 0.5372, Week 10 *p* = 0.0827, Week 14 *p* < 0.0001, Week 18 *p* = 0.0007, Week 22 *p* = 0.0196

On day 0 of the study, the mean level of PCV2 antibodies was 9.2 log_2_ for the vaccinated animals, and 9.1 log_2_ for the controls (mean of all trial piglets was 9.15 log_2_). Serology of PCV2 and M. hyo confirmed the presence of PCV2 and M. hyo field infections. At the time of vaccination, 42 % of the vaccinated piglets and 45 % of controls were seropositive for M hyo. M. hyo serological seroconversion occurred after the 10^th^ week of age. Throughout the study the percentage of M hyo seropositive pigs was higher in the vaccinated groups than in the controls (Fig. 1). Analysis revealed significant differences among groups in samples from weeks 14, 18 and 22 (*p* < 0.0001, *p* = 0.0007, and *p* = 0.0196, respectively).

**Fig. 2:**
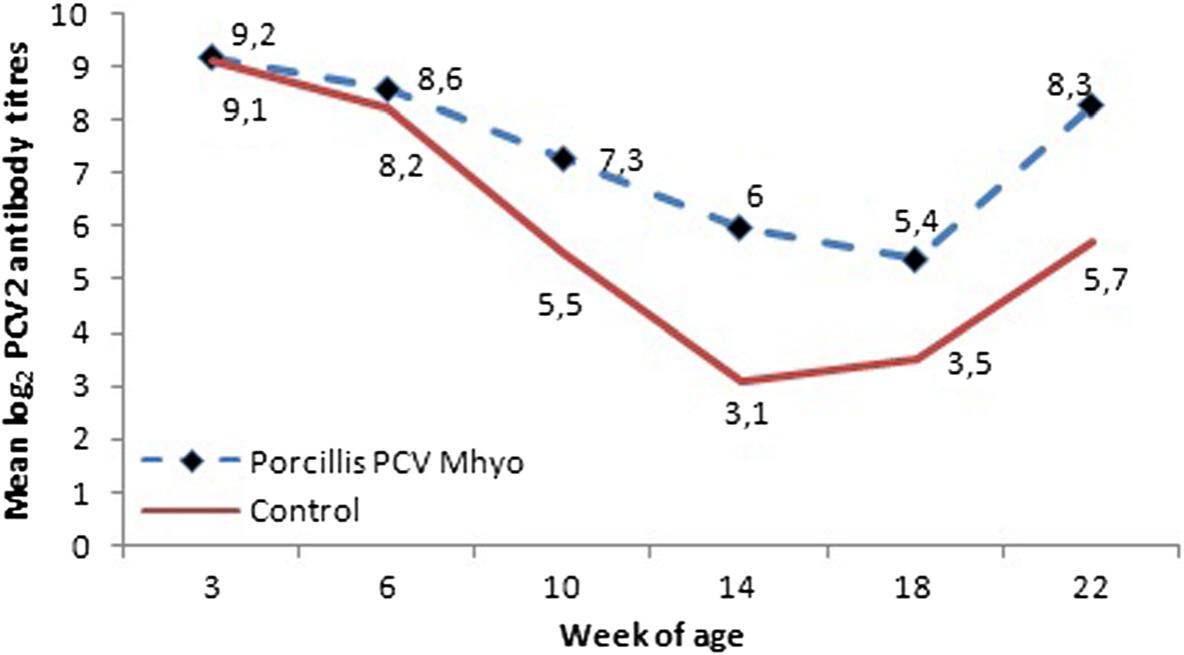
PCV2 serological results, by vaccination group and week of age legend: Statistical differences levels: Week 3: *p* = 0.9162, Week 6 *p* = 0.3069, Week 10 *p* < 0.0001, Week 14 *p* < 0.0001, Week 18 *p* = 0.0013, Week 22 *p* < 0.0001

The mean antibody titres against PCV2 in both groups increased between 18 and 22 weeks of the study, indicating a field infection during the finishing period. The titres in the vaccinated group were higher than in the controls from 6 weeks until the end of the study. The results are summarised in Fig. 2. Statistically significant differences were observed in samples from weeks 10, 14, 18 and 22 (*p* < 0.0001, *p* < 0.0001, *p* = 0.0013, and *p* < 0.0001, respectively).

The vaccinated animals showed a significant reduction in viraemia when compared with controls. The results of quantitative real-time PCR (qPCR) for PCV2 viral DNA (samples from week 18 and 22 of the study) showed that the mean area under the curve [AUC: log_10_ DNA copies PCV2 /per μl DNA extract * week; defined as the sum of segments, each defined by 2 measurement time points in the viraemia plot per subject] was less than one third of the control group (1.47 vs. 5.42; ANOVA *p* < 0.0001). The results, expressed as the mean log_10_ DNA copies per μl DNA extract and the percentage of PCV2 positive samples are summarised in Table 2.

**Table 2:** PCV2 viraemia results, by vaccination group and week of age

Week of age	Porcilis PCV M Hyo	Control
18	0.63 (20 %)	1.14 (28 %)
22	0.35 (14 %)	2.20 (64 %)
AUC^c^	1.47/0.0^a^	5.42/5.36^b^

Mean log_10_ DNA copies PCV2/per μl DNA extract and percentage (in parenthesis) of PCV2 positive serum samples, as well as area under the curve (AUC), by vaccination group and week of age

^c^AUC expressed as log10 DNA-copies *week. AUC results are presented as Mean/Median

One DNA copy/μl DNA is equivalent to 250 DNA copies per ml of serum

Level of significance (ANOVA) among superscripts ab: *p* < 0.0001

Furthermore, with regard to other pathogens tested (samples of 14th, 18th and 22nd week of the study), it was demonstrated that towards the end of the study nearly all pigs were seropositive for PRRS virus and had rising antibody titres against the outer membrane protein (OMP) of APP. Low titres were measured for antibodies against swine influenza viruses (data not shown).

## Discussion

Study results support that the test vaccine administered at 3 weeks of age, can induce a significant immune response against M hyo and PCV2, and can play a major role in improving LLS and ADWG of vaccinated pigs in comparison with non-vaccinated animals. The vaccinations scheme used in this trial successfully induced immunity of piglets as seen by antibody response through the course of the study. Thus, adequate immune priming against M hyo and PCV2 is considered the main factor that lead to better LLS and ADWG in the vaccinated group. Similar results have been shown in other research efforts with one-shot vaccination against each of those pathogens separately [3, 15, 16, 20]. Our results suggest that one-shot vaccination with a ready-to-use combination vaccine against both pathogens at 3 weeks of age significantly benefits the primary parameters investigated in this study, and in a similar way as in separate vaccination.

In addition, PCV2 viraemia results (expressed as AUC) were significantly better in the vaccinated group when compared with controls on both the 18th and the 22nd week of age. Results of increasing PCV2 antibody titres after the 18th week, in both groups, suggest a detectable PCV2 occurrence at a time point around 14 weeks after vaccination. The PCV2 antibody titre alterations (greater mean levels in vaccinated animals when compared with controls), along with strong viraemia reduction during late fattening in vaccinated pigs, clearly suggest a positive impact of the test vaccine for the control of subclinical PCV2.

Due to the nature of the test vaccine, a direct comparison with results from studies in which M hyo and PCV2 vaccines were administered separately cannot be done. On the other hand, the interference of PCV2 MDA in active immunization of piglets after vaccination is discussed as a controversial issue. It has been stated that maternal antibodies can affect the age of PCV2 infection [19] while it has also been demonstrated that high PCV2 MDA levels (≥10 log2) could interfere with piglets' active seroconversion at vaccination without having a negative impact on vaccine efficacy [13]. Nevertheless, a negative MDA effect on humoral immune response of piglets could not be supported by the findings of this study.

Moreover, it could be hypothesized that the largest part of ADWG improvement should be attributed to the effect of vaccination against M. hyo since the trial farm had more severe EP than PCVD. Similarly, differences in body weight at slaughter could also be attributed to better control of EP in vaccinated pigs. Even if those differences on body weight were not statistically significant, they show a noteworthy tendency for improvement of approximately 4.9 kg live weight at slaughter, which is extremely important from a financial standpoint. However a future research effort under field conditions with severe PCVD and less clinically severe EP would be needed to elucidate the effect of each pathogen on ADWG and body weight improvement.

Furthermore, although not in the scope of this study, the use of the test product for immunization of pigs against two significant pathogens can be considered more practical since there is less labour, handling and injections (less stressful for the animals) needed. Additionally, since no local or systemic reactions were observed (data not shown), the test product was considered safe under the conditions used in this study.

## Conclusions

The Porcilis PCV M Hyo combination vaccine was effective for use in piglets at 3 weeks of age. Vaccination resulted in greater percentage of M hyo seropositive pigs and increased PCV2 antibody titres, for the total trial period, as well as significant reduction of the severity of LLS and improvement of ADWG during the finishing and the overall study period, along with significant reduction of the PCV2 viral load.

## Methods

### Trial design

The study was carried out according to a controlled, randomised, and blinded design in a Greek pig herd with a history of M. hyo infection and subclinical PCV2 co-infection. In total 602 healthy 3 week old suckling piglets (study day 0), originating from 4 weekly farrowing batches were allocated randomly, within litters, to one of two groups (vaccinated or control). Control group animals (299 pigs) received saline intramuscular injection on study day 0. At the same time point, the vaccinated group (303 animals) received 2 ml of the test product. All injections were applied intramuscularly in the neck area. The animals included in the study were fed and received water according to standard farm procedures. The sows of which piglets were included in the study were routinely vaccinated against PCV2 (vaccination at 2–3 weeks before parturition), Aujeszky’s disease virus, porcine reproductive and respiratory syndrome virus (PRRSV), porcine parvovirus, *Erysipelothrix rhusiopathiae*, atrophic rhinitis and *Escherihia coli*.

The study was performed in conformity with the requirements of EU note for guidance “Specific Requirements for the production and control of Pig Live and Inactivated Viral and Bacterial Vaccines (III/3362/92)”, Ph.Eur General Chapter 5.2.7 “Evaluation of efficacy of veterinary vaccines and immunosera” and Ph.Eur Monograph 2448 “Porcine enzootic pneumonia vaccine (inactivated)”. The procedures in this clinical study were performed according to the Code of practice for the Conduct of Clinical trials for Veterinary Medical Products [21] and the animals were maintained in accordance with National and European Animal Welfare requirements [22, 23].

### Test product

The test product is a new ready-to-use combination vaccine consisting of PCV2 ORF2 expressed in Baculovirus and inactivated *M. hyo* strain J in Emunade® adjuvant. The present study was done to evaluate field efficacy of this new vaccine in a farm with concurrent clinical M. hyo and subclinical PCV2 infection (both occurring after natural exposure to the pathogens) as confirmed in a pre-trial screening that included lung lesion scoring at slaughter for M. hyo and PCR testing of blood samples for PCV2 (data not shown).

### Parameters

Efficacy parameters were LLS, ADWG, mortality, morbidity, PCV2 viraemia, and pleuritis lesions. To obtain the efficacy data, the pigs were weighed individually at approximately 1 week of age, at transfer to the finishing unit (week 9–10 of age), and before slaughter (week 21–22 of age). Last weighing pre-slaughter was performed in 256 vaccinated pigs and 255 control animals.

The lungs were examined individually at slaughter to assess LLS and pleuritis lesions score according to the method of Goodwin and Whittlestone (1973) [24]. In total, 255 lungs from vaccinated animals and 254 lungs from control pigs were examined. The lungs were scored for typical M hyo. lesions. For each lung lobe, the percentage of the surface with signs of typical M hyo associated inflammation (consolidated, grey to purple coloured) was estimated. These percentages (in fact proportions) were multiplied with the weighing factor of each lobe [24] and added up to obtain the total LLS. Thus, the minimum lung lesion score for an animal was 0 and the maximum 55. Also pleuritis lesions were investigated and scored from 0 to 2 (0 = no pleuritis lesions, 1 = topical adhesions (spots) or 2 = larger adhesions). Medication was recorded and necropsy was performed in pigs that died during the study, unless the cause of death was clear and could not be related to PCV2 or M hyo infection and vaccination (eg crushed suckling piglets), to establish the cause of death, based on gross lesions.

### Blood sampling investigation

Blood samples were collected from 45 piglets per group at regular intervals (3, 6, 10, 14, 18, 22 week of age) and the sera were tested with ELISA for antibodies against PCV2 [25] and M. hyo (Swine HerdChek M. Hyo IDEXX). The blood samples collected at 18 and 22 weeks were tested by qPCR for PCV2 DNA. The blood samples taken at weeks 14, 18 and 22 of the study were also tested for antibodies against APP with the APP OMP ELISA [26], PRRSV (IDEXX PRRS X3 Ab Test), and swine influenza viruses (H1N1, H1N2 and H2N3) by HI test [27].

For PCV2 an in-house ELISA of MSD Animal Health was used as previously described [25]. Titres in the PCV2 ELISA were expressed as the reciprocal of the serum dilution with a calculated extinction value of 50 % maximum extinction. Based on the serological results, the appropriate samples (i.e. time points) were selected for determination of the PCV2 viral load in the serum. The amount of PCV2 genomic DNA was quantified by qPCR as previously described [25]. The results were expressed as log_10_ genome copies/μl DNA extract. One copy/μl DNA extract is equivalent to 250 copies per ml of serum. If the viral load was below the detection limit of 2.00 log_10_ copies/μl DNA extract, the result was considered negative and set at zero (0). The ELISA for M. hyo and PRRS were carried out as recommended by the supplier. The results were expressed as ‘positive’, ‘inconclusive’ or ‘negative’.

### Statistical methods

The pig was the statistical unit and level of significance was set at 0.05. Descriptive statistics (frequency tables, means, standard deviations etc.) were used to summarize results. Specifically, LLS and ADWG comparisons among groups were tested in a mixed model ANOVA (lung lesions log transformed before analysis). For LLS comparisons, the vaccination group was included as fixed effect and the sow and production batch as random effects. For ADWG comparisons the vaccination group and gender with their interaction were included as fixed effects and sow and production batch as random effects. The body weight at one week of age was included in the model as a covariate.

The AUC (Area Under the Curve) was used for the analysis of PCV2 viraemia data. In the viraemia plot per subject, the y-axis represents the viraemia level at a given time point and the x-axis the time (in weeks) after vaccination. The AUC segment defined by 2 measurement time points is therefore expressed as log_10_ DNA-copies of PCV2 per μl DNA extract * week of study. The AUC is calculated by summing the area of these segments over the entire period in which the samples were analysed. The data were ranked before analysis by ANOVA with treatment as factor.

The Cochran Mantel Haenszel method with production batch as classification variable was used for the comparison between groups, as regard to the parameters pleuritis lesions (absent or present) and morbidity.

PCV2 antibody titres were analysed by mixed model ANOVA and the proportion of M hyo positive samples by generalised estimating equation (GEE) followed by LS means to compare the results between the treatment groups per week.
